# P-1331. Effect of Transfusion-Transmitted Infection Pre-Donation Deferrals on Subsequent Donations in a Military Blood Donation Center

**DOI:** 10.1093/ofid/ofae631.1509

**Published:** 2025-01-29

**Authors:** Somin Kwon, Sorana Raiciulescu, Brian Casleton, Glorimar Rivera, Melita Gella, Erin Winkler, Angela Osuna, Theresa Casey, Heather Yun, Joseph Marcus

**Affiliations:** National Capital Consortium Internal Medicine Residency, Bethesda, Maryland; Uniformed Services University Biostatistics Consulting Center, Bethesda, Maryland; Armed Services Blood Bank San Antonio, San Antonio, Texas; Armed Services Blood Bank San Antonio, San Antonio, Texas; Armed Services Blood Bank San Antonio, San Antonio, Texas; BAMC, San Antonio, Texas; BAMC, San Antonio, Texas; BAMC, San Antonio, Texas; Brooke Army Medical Center, San Antonio, TX; Brooke Army Medical Center, San Antonio, TX

## Abstract

**Background:**

To minimize the risk of transfusion-transmitted infections (TTI), all prospective blood donors are required to undergo extensive screening via questionnaire and physical exam. Donors who report behaviors or conditions that may increase risk of TTIs are temporarily deferred from donating. In recent years, significant changes to the deferral policy have been instituted to expand the donor eligibility pool and mitigate the blood supply crisis. In this study, we evaluate the impact of deferring donors at increased risk of TTI on future donation attempts to further inform how these policies may affect the military population.

**Figure:**
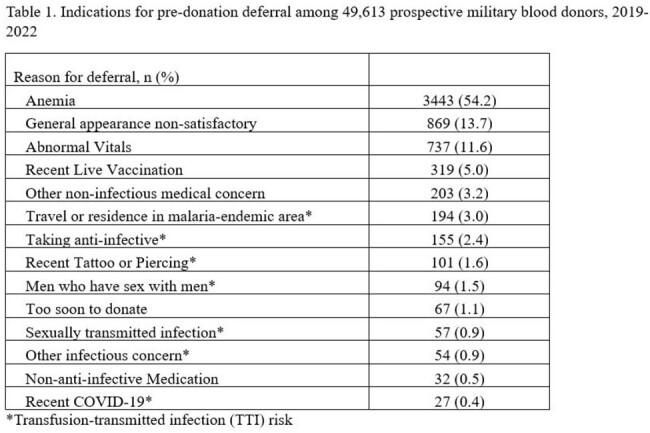

**Methods:**

This retrospective chart review evaluated all blood donors with pre-donation deferrals at a single site between October 2019 to December 2022. Among the fourteen deferral categories, seven were classified as behaviors or conditions that increase risk of TTI including: taking an anti-infective, travel to malaria-endemic area, recent tattoo or piercing, recent sexually transmitted infection (STI), recent COVID-19 infection, man who has sex with men (MSM), or other infectious concern. The rate of donation reattempts within the study period was assessed in donors deferred for TTI risk and compared to that of donors deferred for low hemoglobin matched by sex, age, and BMT status.

**Figure:**
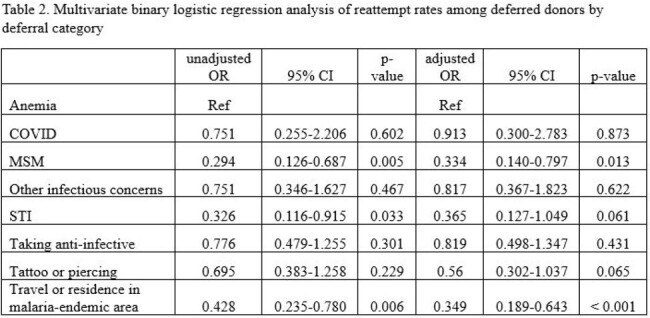

**Results:**

6,352 (12.8%) of the 49,613 blood donors identified during the study had a pre-donation deferral. Over half of all deferrals were due to anemia (54.2%) and approximately a tenth for TTI risk (Table 1). We found that donors who were deferred for TTI risk were significantly less likely to attempt redonation during the study period compared to those deferred for anemia (p< 0.001). On multivariate analysis, donors deferred for MSM and travel or residence in a malaria-endemic area were independently associated with a decreased redonation attempt rate and increased time to reattempt (Table 2, Figure 1).

Time to redonation for deferred blood donors with a TTI risk (dark green) or anemia (light blue).

**Figure:**
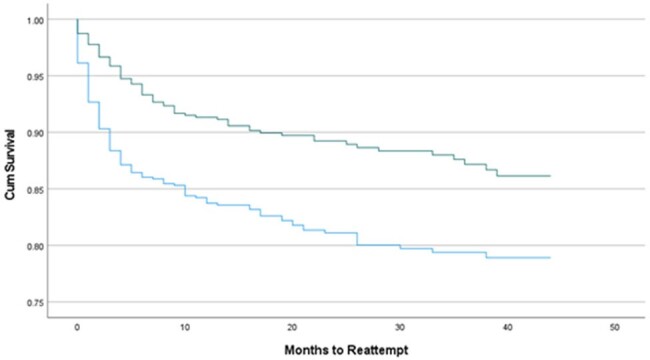

**Conclusion:**

Pre-donation deferrals for TTI risk were associated with a decreased rate of redonation and increased time to reattempt compared to donors deferred for anemia.

**Disclosures:**

**All Authors**: No reported disclosures

